# Methodological aspects of economic evaluations and health economic models in glioblastoma: A systematic literature review

**DOI:** 10.1093/nop/npaf113

**Published:** 2025-10-30

**Authors:** Erika Országh, Judit Józwiak-Hagymásy, Tamás Dóczi, Dóra Mezei, Bertalan Németh, Hédi Varga, Attila Tordai, Tomas Kazda, Thierry Gorlia, Caroline Quoilin, Matthias Preusser, Giuseppe Minniti, Marcell Csanádi

**Affiliations:** Syreon Research Institute, Budapest (E.O., J.J.-H., T.D., D.M., B.N., H.V., M.C.); Syreon Research Institute, Budapest (E.O., J.J.-H., T.D., D.M., B.N., H.V., M.C.); Syreon Research Institute, Budapest (E.O., J.J.-H., T.D., D.M., B.N., H.V., M.C.); Syreon Research Institute, Budapest (E.O., J.J.-H., T.D., D.M., B.N., H.V., M.C.); Syreon Research Institute, Budapest (E.O., J.J.-H., T.D., D.M., B.N., H.V., M.C.); Syreon Research Institute, Budapest (E.O., J.J.-H., T.D., D.M., B.N., H.V., M.C.); Department of Transfusion Medicine, Semmelweis University, Budapest (A.T.); Department of Radiation Oncology, Masaryk Memorial Cancer Institute, Brno (T.K.); European Organisation for Research and Treatment of Cancer (EORTC) Headquarters, Brussels (T.G., C.Q.); European Organisation for Research and Treatment of Cancer (EORTC) Headquarters, Brussels (T.G., C.Q.); Department of Medicine I, Division of Oncology, Medical University of Vienna, Vienna (M.P.); Department of Radiological Sciences, Oncology and Anatomical Pathology, Sapienza University of Rome, Rome (G.M.); Syreon Research Institute, Budapest (E.O., J.J.-H., T.D., D.M., B.N., H.V., M.C.)

**Keywords:** economic evaluations, economic modeling, evidence synthesis, glioblastoma, systematic literature review

## Abstract

**Background:**

Glioblastoma is considered one of the deadliest malignant solid tumors. The disease severity and the treatment intensity place a significant burden on patients, their family and caregivers, and the health systems. This study aimed to review the methodological aspects and quality of economic evaluations and health economic models in glioblastoma with a special focus on Europe and North America.

**Methods:**

A search was performed covering Medline (via PubMed), Embase, Scopus, Cochrane Library, and PROSPERO. Gray literature search and snowball sampling were also conducted. Studies were eligible if they included glioblastoma patients, contained data on economic evaluations, and were in our geographical focus. The protocol was registered in PROSPERO (ID: CRD42023488181).

**Results:**

The review of 4052 records resulted in 19 peer-reviewed articles, 1 health technology assessment agency document, 2 conference posters, and 1 thesis. Most studies investigated pharmaceuticals in combination with radiotherapy (*n* = 7). Five studies focused on tumor-treating fields in combination with maintenance temozolomide. Most studies investigated newly diagnosed patients (*n* = 18). Economic modeling was used in 15 cases; the majority used the "traditional" 3-state Markov cohort or partitioned-survival modeling approach (health states: progression-free, progressed, dead). Input data, particularly for utilities, were outdated in many cases. We identified only 2 studies with societal perspective, and these had limited scope.

**Conclusion:**

Our review identified gaps in the literature related to the economic evaluations on relapsed glioblastoma patients and to comprehensive assessments using a societal perspective. We also concluded that there is a lack of relevant, high-quality, and up-to-date utility data for glioblastoma in the studies.

## Graphical Abstract

**Graphical Abstract:**
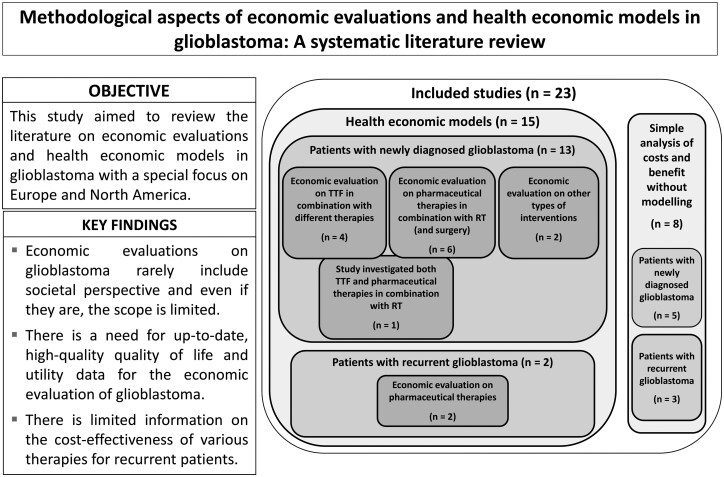

Key Points

- Despite the complex disease burden on patients and beyond, economic evaluations on glioblastoma rarely include societal perspective.
- High-quality and up-to-date quality of life and utility data for glioblastoma are warranted for future evaluations.

Importance of the Study

The latest systematic literature review on the economic evaluation of glioblastoma was published in 2014, which reported an overview of 5 cost-effectiveness studies involving temozolomide (TMZ). Therefore, there was a need to provide an up-to-date summary of the relevant literature. We identified a total of 23 relevant studies; 18 of them presented an evaluation based on an economic model. Reflecting on the developments in the past years, evaluations focused on new therapeutic approaches, such as adding bevacizumab as a concomitant regimen, tumor-treating fields in combination with maintenance TMZ, or various treatment schedules for TMZ. Key findings: despite the huge economic burden on patients, their family, and caregivers, economic evaluations on glioblastoma rarely include a societal perspective; there is a need for high-quality and up-to-date quality of life and utility data for glioblastoma to be used in economic evaluations; limited information is available on the cost-effectiveness of various therapies for recurrent patients.

Glioblastoma, classified as an IDH-wildtype tumor by the WHO, is regarded as one of the deadliest of all malignant solid tumors and is the most frequent primary malignant brain tumor. Initial symptoms may be variable depending on the particular function of the affected brain area.^1^^,^^2^ Initial imaging is performed by magnetic resonance imaging usually indicating unifocal alterations with ring-like contrast enhancement reflecting an abnormal vascularization and tendency to spontaneous central necrosis.^3^ The age at diagnosis, performance status (eg, Karnofsky Performance Status), and extent of resection are the most important prognostic factors affecting overall survival (OS).^4^ Currently, MGMT promoter methylation status is the most significant molecular prognostic and predictive attribute, and is relevant as a biomarker of sensitivity to alkylating ­chemotherapy. The median OS of glioblastoma patients in population-based studies is approximately 10-12 months, while for patients 65 years and older, it is approximately 6 months.^5^^,^^6^

The cornerstone of first-line glioblastoma management is surgery, with a recommended maximal safe resection consistent with the preservation of neurologic function. Adjuvant radiation therapy (RT) is a standard component of glioblastoma therapy, and systemic adjuvant chemotherapy has consistently been the third modality.^3^^,^^7^ The EORTC 26981/22981/CAN-NCIC-CE3 trial established a new standard of care based on chemo-radiotherapy following surgery, as it showed the superiority of temozolomide (TMZ) given concomitantly with radiotherapy versus radiotherapy alone.^8^ An alternative to TMZ is the oral nitrosourea-type lomustine (LOM), which has been more widely used in the EU.^9^ The combination of TMZ and LOM can be an alternative for newly diagnosed glioblastoma patients with methylated MGMT promoter.^10^ Tumor-treating fields (TTFs), a non-invasive anticancer therapeutic modality, are considered another treatment option for newly diagnosed glioblastoma based on a recent large-scale phase 3 randomized trial and subsequent real-world data.^11^ Second-line treatment options at disease progression or recurrence are highly dependent on patient performance status (capable vs incapable of self-care), age, and biological nature of the tumor. The decision on providing further active therapy or opting for best supportive care must be decided based on the above-mentioned factors.

The severity of the disease and the treatment intensity place a significant economic burden on the patients, their family and caregivers, and the health systems.^12^ Therefore, it is essential to complement efficacy and safety data with analyses of the economic consequences of therapeutic options. To estimate the financial impact of new treatment alternatives for payers and policymakers, economic evaluations employing various modeling approaches are utilized and are widely accepted in healthcare decision-making.^13^ These analyses provide critical insights into the potential costs and benefits of novel cancer treatments and therapy sequences relative to the standard of care.^14^ Additionally, economic evaluations incorporate quality-of-life considerations through patient-reported outcomes, promoting a patient-centered approach in healthcare decision-making.^15^

Comprehensive reviews of economic evaluations across various oncology disciplines are warranted to understand trends in health economics evidence generation, identify evidence gaps, and supplement existing analyses on specific therapeutic interventions.^16^ In glioblastoma research, the latest review was published in 2014, which reported an overview of 5 cost-effectiveness studies involving TMZ.^17^ Therefore, an updated synthesis of the relevant literature is warranted. This study aimed to review the methodological aspects and quality of economic evaluations and health economic models related to glioblastoma with a special focus on studies from Europe and North America to inform future research and healthcare decision-making in this field.

## Methods

A systematic literature review was conducted, which included both economic evaluations and health economic models with a special focus on glioblastoma. An explorative, preliminary literature search in this field indicated that the literature search should be extended to glioma. The PICOS criteria used to define the research scope are included in the online supplementary Appendix I. Title and abstract screening, full-text screening, and data extraction for each record were performed independently by 2 researchers and reported in accordance with the PRISMA 2020 Statement.^18^ The systematic review protocol was registered in PROSPERO, an open-access, international database of prospectively registered systematic reviews.^19^ Reference number: PROSPERO 2023 CRD42023488181.

The search was performed covering the following databases: Medline (via PubMed), Embase, Scopus, Cochrane Library, and PROSPERO. Time limit was not applied for the literature search. Searches were performed on August 23, 2023. However, to provide a more up-to-date presentation of the literature, a search update was conducted in September 2024 using the same search strings and the same databases. Relevant publications were included in the discussion section of this manuscript. Search terms were defined according to the disease area (eg, glioblastoma or glioma) and the outcomes of interest (eg, parameters of cost-effectiveness analysis). The search strings and the number of hits for each database are presented in online supplementary Appendix II. Records retrieved from the databases were imported into Covidence^20^ for screening. Search hits were first automatically de-duplicated, then further duplications were identified manually. To increase the sensitivity of the systematic review, backward and forward snowball searches were also conducted with the Citationchaser software.^21^ The backward search of formerly published review articles was done manually. Snowball search results underwent the same screening and data extraction processes as articles from other sources.

Title and abstract screening, as well as full-text screening phases, included tailored training for team members and a pilot exercise in which a limited sample of studies were randomly selected and reviewed. Studies were considered eligible and selected for inclusion if they involved patients with glioblastoma or glioma and contained data related to economic evaluations, with a geographical focus on Europe and North America. No restrictions were applied regarding treatment patterns or the date of publication. Full-text studies written in English were eligible for data extraction. Further details on the eligibility criteria used to select relevant publications can be found in online supplementary Appendix III.

All data relevant to the review topic were extracted from papers after the literature screening. Data extraction was conducted using Excel tables. A pilot data extraction template was developed, and extraction was performed on a selection of example studies chosen at random. Following the pilot extraction, the data extraction template was finalized based on the comments and feedback from the extractors. All extracted data were double-checked by a second reviewer for quality assurance. When it was necessary, conflict resolution in data extraction was ensured by the project leader. The reviewers were not blinded to journal and author details; however, no conflict of interest emerged during this work. Data items that were collected are listed in online supplementary Appendix IV. A narrative synthesis of the collected information was performed.

Since evidence not published in peer-reviewed journals can contribute to the comprehensiveness of a systematic review, gray literature sources were included in the search strategy. To find relevant health economic evaluations and models related to glioblastoma or glioma, databases of relevant health technology assessment (HTA) agencies, along with conference databases, were searched. The list of reviewed gray literature sources is included in online supplementary Appendix V.

Quality assessment of peer-reviewed articles was performed to evaluate the methodological quality of the original publications of economic evaluations. Studies with limited information (ie, conference posters) and materials from HTA agencies that were mostly appraisals were excluded from the quality assessment. The methodological quality of the included health economic studies was evaluated using the ECOBIAS checklist.^22^ This tool was developed to conduct a risk-of-bias assessment in model-based economic evaluations. It includes an overall checklist and more specific aspects focusing on bias related to structure, data, and consistency, with a total of 22 items.

## Results

### Literature Search Results

A total of 3631 records were identified through 4 databases (Medline, Embase, Scopus, and Cochrane reviews), and further 1777 hits were included as the result of the snowball search. During snowball sampling, a thesis with a relevant topic was identified; since no related scientific publication was found, the thesis was imported to Covidence for data extraction. Out of the total records, 1356 were duplicates, the majority of which originating from the snowball search. In the remaining 4052 screened records, 3842 met at least 1 exclusion criterion during title and abstract screening; therefore, 210 articles were eligible for full-text screening. During this stage, 167 articles were excluded, and 42 peer-reviewed articles and 1 thesis work were considered relevant by applying the inclusion criteria for glioma. The gray literature review identified 1 HTA agency document and 4 ISPOR posters as relevant by applying the inclusion criteria for glioma. In total, 48 studies were included in the final analysis.

**Figure 1 PRISMA chart of the systematic literature review:**
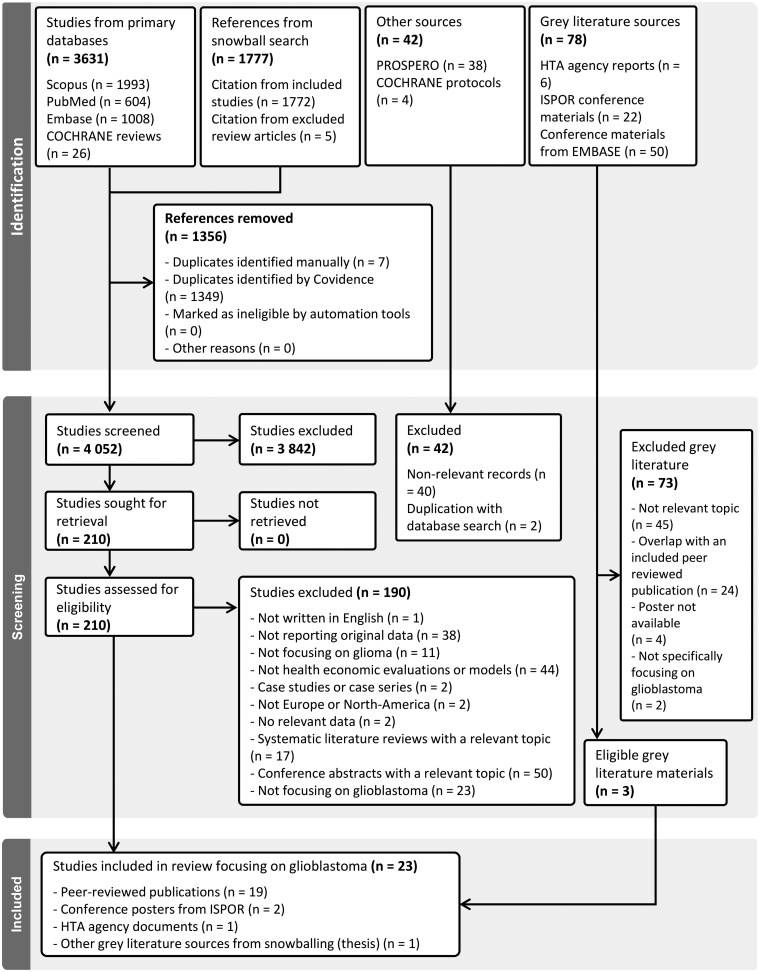
.

In this current review, we focus specifically on glioblastoma patients and provide an overview of the economic evaluations. Of the 48 identified studies, 22 exclusively included patients with glioblastoma, and 25 investigated patients with other types of gliomas. Additionally, 1 study incorporated 2 separate models, 1 for glioma and 1 for glioblastoma. Among the 23 studies involving glioblastoma patients, 18 focused on newly diagnosed cases and 5 investigated patients with recurrent disease. Figure 1 summarizes the process of search and screening phases related to the health economic evaluations and models on glioma.

**Figure 2 Graphic representation of the included studies according to modelling approach, patient population and type of intervention:**
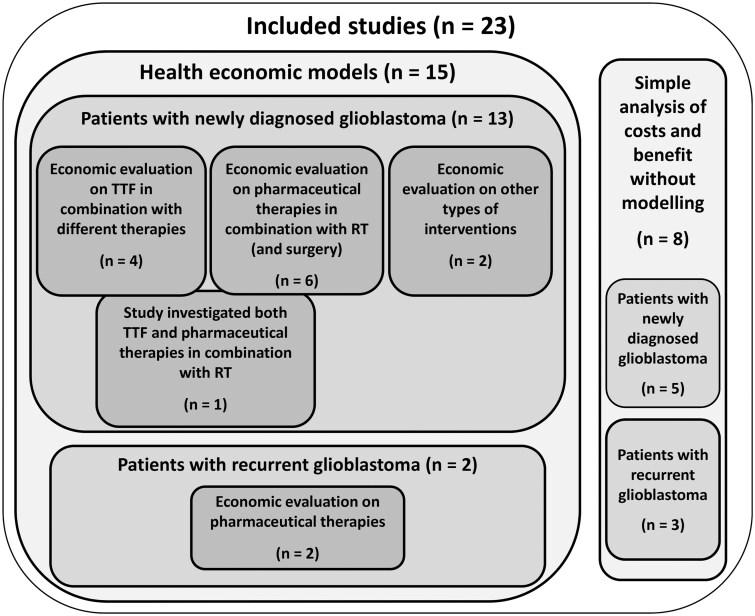
.

Among the 23 studies involving glioblastoma patients, 15 included details on health economic models, while 8 reported cost and benefit calculations based on observational studies or clinical trials without economic modeling. The distribution of the included studies by modeling approach, patient population, and intervention type investigated is shown in Figure 2. This manuscript focuses on the studies with health economic models, while those without modeling are included in online supplementary Appendix VI.

### Summary of Economic Evaluation Studies With Economic Modeling on Pharmaceutical Therapies in Combination With Radiotherapy

We identified 7 studies^23–29^ focusing on pharmaceutical therapies in combination with radiotherapy (Table 1). All studies involved newly diagnosed glioblastoma patients with similar characteristics, although 1 study^23^ specifically focused on elderly patients. One study was conducted in the United Kingdom,^28^ another in Germany,^29^ 3 in the United States^23^^,^^24^^,^^27^, and 3 in Canada.^24–26^

**Table 1.** Studies investigating pharmaceutical therapies in combination with radiotherapy

Reference	Study country	Investigated vs comparator therapy	Modeling approach	Health states	Modeling timeline	Modeling perspective	Study outcome	Patient population
Chen et al. (2021)^23^	United States^a^	**TMZ + RT** vs RT	Decision tree combined with partitioned survival model	SD; PD; Death	5 years	Health care system	Δ Cost/Δ QALY	Patients with newly diagnosed GB [WHO grade IV astrocytoma] aged 65 years or older
Connock et al. (2021)^24^	United States	**TMZ + RT** vs RT	Partitioned survival model	SD; PD; Death	5 years	Societal perspective	Δ Cost/Δ QALY	Patients with newly diagnosed, histologically confirmed glioblastoma^b^
	Canada	**Bevacizumab + TMZ + RT** vs Placebo + TMZ + RT	Partitioned survival model	SD; PD; Death	2 years	Health care system	Δ Cost/Δ QALY	Patients with newly diagnosed glioblastoma^c^
Fischer (2016)^25^	Canada	**Bevacizumab + TMZ + RT** vs TMZ + RT + placebo	Markov cohort model	PFT; TP; Death	5 years	Health care system	Δ Cost/Δ QALY	Patients with newly diagnosed glioblastoma^c^
Kovic and Xie (2015)^26^	Canada	**Bevacizumab + TMZ + RT** vs TMZ + RT	Markov cohort model	PF; PD; Death	2 years	Health care system	Δ Cost/Δ QALY	Patients with newly diagnosed glioblastoma^c^
Messali et al. (2013)^27^	United States	**TMZ + RT** vs RT	Markov cohort model	SD; PD; Death	5 years	Societal perspective	Δ Cost/Δ QALY	Patients with newly diagnosed, histologically confirmed glioblastoma^b^
NICE (2007)^28^	United Kingdom	**TMZ + RT** vs RT	Markov cohort model	Surgery; Postoperative recovery; Radiotherapy; SD; PD; Death	5 years	Health care system	Δ Cost/Δ QALY	Patients with newly diagnosed glioblastoma^b^
Waschke et al. (2018)^29^	Germany	**Open-ended long-term TMZ + RT** vs 6 cycles of adjuvant of TMZ + RT	Markov cohort model	SD; PD; Death	5 years	Health care system	Δ Cost/Δ quality-adjusted life-month Δ Cost/Δ QALY	Patients with newly diagnosed, histologically confirmed glioblastoma^b^

a The analysis was also performed for China.

b The study had a patient population with the same characteristics as those in the EORTC-NCIC trial: aged 18-70 years with newly diagnosed and histologically proven glioblastoma (WHO grade IV astrocytoma), with a WHO performance status of 0-2 and adequate hematological, renal, and hepatic function.^8^

c The population was similar to the AVAglio trial^30^ and the RTOG 0825 clinical trial^31^ and included adults (≥18 years old) of both sexes with newly diagnosed glioblastoma after biopsy or resection, with WHO performance status of 0-2 or Karnofsky performance status ≥70; adequate hematologic, hepatic, and renal function; and acceptable blood coagulation levels.

Abbreviations: PD, progressed disease; PF, progression-free; PTF: progression-free tumor; QALY: quality-adjusted life years; QALY: quality-adjusted life years; RT: radiotherapy; SD: stable disease; TMZ: temozolomide; TP: tumor progression.

Four studies investigated TMZ + RT compared to RT,^23^^,^^24^^,^^27^^,^^28^ while 1 study^29^ compared different regimens of TMZ in combination with RT. These studies shared the same patient population based on the largest RTC (study ID: EORTC 26981/22981 or CAN-NCIC-CE3), which randomized 573 newly diagnosed, histologically confirmed glioblastoma patients from 85 institutions across 15 countries who received either RT (286 patients) or RT plus TMZ (287 patients).^8^ This RTC was also used to calculate transition probabilities in some studies.^24^^,^^27^^,^^29^ Out of the 5 TMZ studies, 4 studies applied 3 health states—stable disease (SD), progressive disease, and death^23^^,^^24^^,^^27^^,^^29^—while 1 study included 7 health states.^28^ A healthcare system perspective was applied in 3 models,^23^^,^^28^^,^^29^ whereas studies by Messali et al.^27^ and Connock et al.^24^ used a societal perspective, including caregiver time as an indirect cost.

Three Canadian studies compared bevacizumab + TMZ + RT to TMZ + RT.^24–26^ All studies had the same patient population based on the largest RTCs, the AVAglio trial,^30^ and the RTOG 0825 trial,^31^ which included newly diagnosed, histologically confirmed glioblastoma patients, with WHO performance status ≤2 or Karnofsky performance status ≥70, and adequate hematologic, hepatic, and renal function.^30^^,^^31^ These RTCs were also used to calculate transition probabilities in 3 studies.^24–26^ The studies had 3 health states: progression free (tumor) or SD, progressed disease (PD) or tumor progression, and death.^24–26^ Two studies used a Markov cohort model,^25^^,^^26^ while 1 study utilized a partitioned survival model.^24^ All studies adopted a healthcare system perspective, with a time horizon of 2 years in 2 studies^24^^,^^26^ and 5 years in 1 study.^25^

All included studies reported the incremental cost per incremental quality-adjusted life-years (QALYs), while 1 study also reported incremental cost per quality-adjusted life-month.^29^ In 3 studies,^26^^,^^28^^,^^29^ a decrease of 0.02 QALYs per consecutive month spent in the progression state was applied, translating to a total annual decrease of 0.24 QALYs.

In the study by Connock et al.,^24^ the authors selected 3 previously published time-invariant Markov models (TIMMs) and compared their outcomes with those of partitioned survival models, utilizing the same data sources, utility values, time horizons, cycle times, and annual discount rates specific to each TIMM. Two of the original Markov models were the studies conducted by Messali et al.^27^ and Kovic and Xie,^26^ as previously described. The authors assumed that the TIMMs provided less realistic estimates of costs and benefits, primarily due to the rapid depletion from the SD state and/or accumulation in the dead state.

There was 1 study related to technology appraisal done by an HTA agency (NICE); therefore, this study had a critical review approach instead of traditional original research methodology. The study by NICE^28^ included the following health states: symptomatic glioblastoma, surgery, postoperative recovery, radiotherapy, SD, progressive disease, and death. The authors assumed that patients in the “progressive” state would experience a constant decline in their quality of life; therefore, they applied a decrement at a rate of 0.5% per model cycle.

The quality assessment included in online supplementary Appendix VII identified 1 significant methodological issue: 1 study did not mention discounting,^29^ despite having a 5-year time horizon. No other major bias was identified. Most studies adopted a healthcare payer perspective rather than a broader societal view, except for Messali et al.^27^ and the related analysis by Connock et al.,^24^ which explicitly adopted a societal perspective. Intermittent data collection bias was a challenge from the assessment point of view, as all papers utilized retrospective resource use data; however, we considered the approach satisfactory if there was no evidence of spot-sampling or leaving out relevant elements of resource use. Sponsor bias was considered adequate if the paper clearly reported on sponsorship and/or conflict of interest. Overall, only Connock et al.^24^ failed to provide explicit disclosure regarding sponsorship or potential conflicts of interest.

### Summary of Economic Evaluation Studies With Economic Modeling on TTFs in Combination With Maintenance TMZ

Among the included studies, 5 investigated TTFs in combination with maintenance TMZ (see Table 2). Four studies were conducted in France and 1 in the United States. All studies adopted a healthcare system perspective and involved a similar patient population of newly diagnosed glioblastoma patients based on the largest RTC (EF-14 trial).^36^ This RTC included adult patients with newly diagnosed glioblastoma with a Karnofsky scale ≥70 who underwent a biopsy or surgery (with or without Gliadel wafers), followed by RT in combination with TMZ chemotherapy. The EF-14 trial enrolled 695 patients across the United States, Canada, Europe, Israel, and South Korea. Patients received maintenance treatment with either TTF plus TMZ (*n* = 466) or TMZ alone (*n* = 229). The results of the EF-14 trial^36^^,^^37^ were used to derive transition probabilities in all included studies investigating TTF.^24^^,^^32–35^

**Table 2.** Economic evaluation studies investigating tumor treating fields in combination with maintenance TMZ

Reference	Study country	Investigated vs comparator therapy	Model type	Health states (if relevant)	Modeling time horizon	Modeling perspective	Study outcome	Patient population
Bernard-Arnoux et al. (2016)^32^	France	**TTF** + **maintenance TMZ vs** maintenance TMZ	Markov cohort model	SD; PD; Death	Lifetime	Health care system	Δ Cost/Δ LYG	Patients with newly diagnosed glioblastoma^b^
Connock et al. (2019)^33^	France	**TTF** + **maintenance TMZ** vs maintenance TMZ	Partitioned survival model	Alive not-progressed (SD); Alive progressed (PD); Death	20 years	Health care system	Δ Cost/Δ LYG; Δ Cost/Δ QALY	Patients with newly diagnosed glioblastoma^b^
Connock et al. (2021)^24^	France	**TTF + maintenance TMZ** vs maintenance TMZ	Partitioned survival model	Alive not-progressed (SD); Alive progressed (PD); Death	12.5 years	Health care system	Δ Cost/Δ LYG	Patients with newly diagnosed glioblastoma^b^
Nino de Rivera et al. (2023)^a^ ^,34^	France	**TTF + maintenance TMZ** vs maintenance TMZ	Partitioned survival model	Alive not-progressed (SD); Alive progressed (PD); Death	20 years	Health care system	Δ Cost/Δ LYG; Δ Cost/Δ QALY	Patients with newly diagnosed glioblastoma^b^
Guzauskas et al. (2019)^35^	United States	**TTF + maintenance TMZ** vs maintenance TMZ	Partitioned survival model	SD; PD; Death	Lifetime	Health care system	Δ Cost/Δ LYG; Δ Cost/Δ QALY	Patients with newly diagnosed glioblastoma^b^

a Conference poster available only.

b The study had a patient population with the same characteristics as those in the EF-14 trial: adult patients with newly diagnosed grade IV astrocytoma, were progression-free after having undergone maximal safe debulking surgery when feasible or biopsy, and had completed standard concomitant chemoradiotherapy with temozolomide, had a Karnofsky Performance Status score ≥70, with adequate bone marrow, liver, and renal function.^36^

Abbreviations: LYG, life years gained; PD: progressed disease; QALY: quality-adjusted life years; RT: radiotherapy; SD: stable disease; TMZ: temozolomide; TTF: tumor treating fields.

All included studies investigated TTF in combination with maintenance TMZ compared to maintenance TMZ alone.^24^^,^^32–35^ Regarding the modeling approach, 2 methods were employed: 4 studies developed partitioned survival models to simulate patient outcomes. Three of them had a 10-year period,^24^^,^^33^^,^^34^ and 2 applied a lifetime horizon.^32^^,^^35^ All models used the health states of SD, PD, and death. The Markov cohort model approach was used in 1 study^32^ with a lifetime horizon. Across all studies (*n* = 5), the primary reported outcome was the incremental cost per life years gained (LYG), with 2 studies also reporting the incremental cost per incremental QALY as well.

In the study of Connock et al.,^24^ the authors compared 2 modeling approaches (Markov cohort vs partitioned survival modeling) across 3 case studies. One of the original Markov cohort models was the study described above by Bernard-Arnoux et al.^32^ The partitioned survival model used the same 3 health states as the original Markov models: alive not-progressed, alive progressed, and dead. In this case, extrapolation beyond the observed survival was necessary. In the case of the TTF models, the Markov model of Bernard-Arnoux et al.^32^ produced a higher ICER value (expressed as cost per LYG) than the partitioned survival model by Connock et al.^24^ It was assumed that the TIMMs provided less realistic estimates for costs and benefits due to the rapid depletion from the SD state and/or accumulation in the dead state.

The studies concluded that although the application of TTF could lead to an increased life expectancy for newly diagnosed glioblastoma patients, due to the high cost of the TTF device, the therapy is not cost-effective,^32^^,^^33^ only within some range of wilingness-to-pay thresholds.^34^^,^^35^

No significant methodological issues were found in the studies, though some minor issues can be mentioned (online supplementary Appendix VIII). The model structures were adequately chosen and in line with patient pathways. All papers adopted a healthcare payer perspective. One study did not provide evidence that variables were checked for double-counting.^34^ Ordinal ICER bias was handled effectively across the majority of the papers, with QALYs commonly used as a cardinal scale for measuring outcomes. However, 2 studies reported only cost/LYG.^24^^,^^32^ The decision to use life-years rather than QALYs was justified given the lack of relevant utility data for glioblastoma. Only Connock et al.^24^ failed to provide explicit disclosure regarding sponsorship or potential conflicts of interest.

### Summary of Economic Evaluation Studies With Economic Modeling on Pharmaceutical Therapies in Recurrent Glioblastoma

We identified 2 studies focusing exclusively on chemotherapies (Table 3). One study was from Spain^38^ and 1 from Finland.^39^ Although both studies had a similar patient population with recurrent glioblastoma, they applied different modeling approaches.

**Table:** **Table 3.**Economic evaluation studies focusing on pharmaceutical therapies in recurrent glioblastoma

Reference	Study country	Investigated vs comparator therapy	Modeling approach	Health states	Modeling timeline	Modeling perspective	Reported outcome	Patient population
Garcia Lopez et al. (2014)^a^ ^,38^	Spain	**Bevacizumab** vs SCP; **Fotemustine** vs SCP; **Extended-dose TMZ** vs SCP	Markov cohort model	Alive without progression; alive with toxicity; progression (absorbing state)	1 year	Health care system	Δ Cost/Δ year to obtain 6 months PFS with stable health state utility value	Patients with recurrent glioblastoma after standard adjuvant treatment based on Stupp’s regimen^b^
Martikainen et al. (2005)^39^	Finland	**TMZ** vs PCV	Markov simulation model	PF; PD; Death	Lifetime	Societal perspective^c^	Δ Cost/Δ life-month; Δ Cost/Δ progression-free months; Δ Cost/Δ QALY	Patients with glioblastoma who underwent primary treatments such as surgery and RT and relapsed

a Conference poster available only.

b Reference Stupp et al.^8^

c A societal perspective was assumed, cost of traveling per visit was taken into account.

Abbreviations: PCV, procarbazine + lomustine + vincristine; PD, progressed disease; PF, progression-free; PFS: progression-free survival; QALY: quality-adjusted life years; RT: radiotherapy; SCP: standard clinical practice; TMZ: temozolomide.

In the poster presentation by Garcia Lopez et al.,^38^ the cost-effectiveness of treatment options for patients with recurrent glioblastoma was investigated. They compared bevacizumab, fotemustine, and extended-dose TMZ to carmustine, which was considered the standard clinical practice. A Markov model was developed with 3 health states: alive without progression, alive with toxicity, and progression (absorbing state). Two-month cycles were used with half-cycle correction. The ICER was expressed as the cost per year to obtain 6 months of progression-free survival (PFS) and the cost per year to obtain 6 months of PFS with a stable health state utility value.

Martikainen et al.^39^ compared TMZ to the procarbazine + LOM + vincristine combination, using a Markov cohort model with 3 health states: progression-free, progression, and death. A lifetime horizon and 1-month cycle length were applied. A societal perspective was assumed that included the cost of traveling per visit. Cost per life-month gained, cost per progression-free life-month, and cost per QALY were calculated.

The quality assessment of the studies identified only minor issues (online supplementary Appendix IX). In the case of Garcia Lopez et al.,^38^ the perspective was not clearly defined, but a societal perspective was assumed, since the cost of traveling per visit was taken into account. The time horizon was 1 year in 1 study,^38^ which is acceptable given the rapid progression of recurrent glioblastoma. In the study of Martikainen et al.,^39^ QoL estimates were gathered using proxy respondents. Utility scores were obtained using a visual analogue scale method. The questionnaires were sent to a limited number of leading neuro-oncologists (*n* = 8), of whom 6 responded. The methods of data identification were mostly transparent; however, in 1 case, the source of some data was not clearly stated, likely due to the limited form of the poster presentation.^38^

### Summary of Economic Evaluations on With Economic Modeling on Other Types of Interventions

We identified 2 studies focusing on other interventions or impacts (Table 4). One study evaluated diagnostics for glioblastoma patients,^40^ while 1 study investigated the impact of insurance status on inpatient hospital costs associated with the first resection in patients with newly diagnosed glioblastoma.^41^ One study was conducted in Belgium,^40^ and the other in the United States.^41^ Both studies employed a decision tree model design.

**Table 4.** Studies investigating other types of interventions

Reference	Study country	Investigated technologies	Model type	Health states (if relevant)	Modeling time horizon	Modeling perspective	Study outcome	Patient population
Baguet et al. (2019)^40^	Belgium	**Follow-up [18F] FET PET vs MRI**	Decision tree cohort model	Responder; Real responder; non-responder; real non-responder; non-real responder; non-real non-responder; false responder; false non-responder	No data	Health care system	Δ Cost/Δ identified non-responder to follow-up treatment	Patients with newly diagnosed glioblastoma who underwent resection
Chandra et al. (2019)^41^	United States	Medicaid group vs non-Medicaid group (Medicare and private insurance)	The model type is not specified, but it is assumed to be a decision tree cohort model.	No data	No data	Hospital	Δ Cost/Δ QALY	Patients with newly diagnosed glioblastoma undergoing their first resection

Abbreviations: 18F-FET PET: O-(2-18F-fluoroethyl)-L-tyrosine Positron Emission Tomography; LYG: life years gained; MRI: magnetic resonance imaging; QALY: quality-adjusted life years.

The study of Baguet et al.^40^ reported the incremental cost per identified non-responder to follow-up treatment and found that [18F] fluoroethyl-L-tyrosine ([18F]-FET) positron emission tomography (PET) is a valuable, cost-effective tool for predicting treatment responses in glioblastoma patients undergoing TMZ maintenance. The results accurately predict clinical outcomes, supported by robust data affirming the reliability of this conclusion.

One study reported incremental cost/QALY gained as an outcome,^41^ conducted from the hospital’s perspective, and concluded that there are differences in surgical costs, hospital stays, survival, and QALY scores based on insurance status in the United States.

The quality assessment of the studies revealed some minor issues (online supplementary Appendix X). In the study by Baguet et al.,^40^ the time horizon was not clearly defined, discounting was deemed irrelevant, and utility weights were not incorporated, which was appropriate given the study’s focus on the cost-effectiveness of follow-up [18F] FET PET scans performed on glioblastoma patients after surgery. In the other study, several items of the ECOBIAS were not applicable due to the short-term hospital stay focus; for example time horizon and discounting were irrelevant, while structural assumptions and treatment effects could not be assessed since no formal economic model or specific treatment comparisons were made.^41^ Additionally, sensitivity analyses to explore uncertainty were not conducted in this study.^41^

## Discussion

Before this systematic review, there was 1 similarly comprehensive review published in 2014 that reported an overview of 5 cost-effectiveness studies involving TMZ either in combination with RT for newly diagnosed patients or in second line alone.^17^ Therefore, there was a need to provide an up-to-date summary of the relevant literature. Reflecting on the developments in the past years, our study identified economic evaluations on new therapeutic approaches; adding bevacizumab as a concomitant regimen, TTF in combination with maintenance TMZ or various treatment schedules for TMZ (eg, fixed cycles or open-ended). Overall, the identified studies had good quality without major methodological problems. The 2 most notable issues were related to the reliability and validity of applied quality of life data and the proper consideration of social perspective in the analyses.

Most models included patients with newly diagnosed glioblastoma, with studies on recurrent glioblastoma patients being underrepresented. These health economic models primarily adopted a healthcare system perspective and utilized various time horizons tailored to the patient population and the therapy. For newly diagnosed glioblastoma patients, the most common time horizon was 5 years for pharmaceutical therapies in combination with RT, while studies investigating TTF + maintenance TMZ often employed either a lifetime or 10-year time horizon. In the 2 studies assessing therapies with recurrent glioblastoma patients, the time horizon was either lifetime or 1 year, which is appropriate given the rapid progression of the disease. The modeling methodologies were generally simplified and uniformly adopted across various jurisdictions. In most cases, Markov cohort models or partitioned survival models were applied, using the traditional approach for health states (ie, progression-free, PD, and death).

Our review indicates that there is a lack of relevant, high-quality, and up-to-date utility data for the economic evaluation of glioblastoma in the literature, with most studies relying on a single source published in 2007,^42^ which estimated utility values from 36 NHS Value of Health Panel experts, using the standard gamble technique.^42^ This study also applied a decrement rate of 0.5% per week during the progressive state in their model to account for the continuously deteriorating quality of life of the patients. The same reduction rate was used in one of the studies that was included in our review.^28^ Conversely, other studies assumed a 0.02 QALY decrease per consecutive month of progression, translating to a total decrease of 0.24 QALYs per year,^26^^,^^29^ underscoring how different assumptions can substantially influence cost-effectiveness outcomes.

Some studies investigated the impact of different modeling approaches, such as Markov models with constant or variable transition probabilities.^29^ In the study of Waschke et al.,^29^ the time-variant transition probabilities of the first model were transformed from monthly OS and PFS data, while in the second model, the median OS and PFS were transformed into constant transition probabilities. Another example compared TIMMs to partitioned survival analysis.^24^ In Markov models, the transition probability can be time-independent or time-varying, but it is always determined by what happens in a given cycle, based on what the state of the patient was in the previous cycle. On the other hand, partitioned survival models are based on the survival curves, and they do not depend in this sense on the previous cycle, but the distributions determine how many patients are alive or have not progressed at a given time. In both cases, the authors came to the conclusion that Markov models with time-invariant/constant transition probabilities provided less realistic cost-and-benefit estimates due to the rapid depletion from the SD state and/or accumulation in the dead state, thus not reflecting accurately and realistically the actual conditions.^24^^,^^29^

Although the majority of the economic models applied a healthcare payer’s perspective, there were some studies that used a societal perspective; in 2 cases, the cost of transport was included in the calculations.^29^^,^^39^ Another example was the study of Messali et al.^27^ in which caregiver time was included as an indirect cost. However, these evaluations were not comprehensive enough, as numerous other indirect costs may occur that were not being accounted for. These require further investigations into key areas of indirect costs, including lost income due to illness, non-medical costs associated with the disease such as informal care, and the broader impact on family members. Due to the rapid deterioration of the quality of life, it is reasonable to explicitly include indirect costs for the evaluations in the future.

Regarding the geographical coverage of the included economic evaluations, certain European regions appear underrepresented, with none of the identified studies conducted in Central and Eastern European countries. According to the literature, while cancer mortality is declining in Western Europe, this trend is not observed in Central and Eastern Europe.^43^ Additionally, these regions allocate considerably less funding to oncology drugs compared to Western Europe, underscoring the urgent need for improved resource allocation, better organization, and the implementation of evidence-based strategies to address disparities.^43^

It is important to highlight that developing a high-quality economic model that appropriately reflects on patient care of glioblastoma is challenging, as treatment options, especially in disease progression or recurrence, are highly dependent on patient performance status (capable vs incapable of self-care), age, and biological nature of the tumor. Additional factors to be considered include the length of prior remission, the nature of the primary tumor (unifocal or multifocal), and if a prior low-grade tumor occurred in the patient’s medical history. Simulating the treatment of recurrent glioblastoma is even more challenging than primary glioblastoma due to its increased malignancy and resistance to standard treatments like TMZ and RT.^44^ The lack of clear, consistent treatment guidelines adds to the difficulty, as many regimens have limited efficacy in clinical trials. On top of that, the high cost of antitumor drugs further complicates the management of recurrent glioblastoma.^44^ The decision on providing further active therapy or opting for best supportive care must be decided on an individual basis. The LEGATO trial (NCT05904119) is investigating the combination of LOM and radiotherapy for progressive glioblastoma.^45^ This ongoing study is the first to include a health economics analysis as early as possible to assess the economic value of the therapies, supporting evidence-based policymaking. This innovative approach could serve as a model for both small and large international studies, facilitating adaptation of country-specific economic models and enabling more comparable policy decisions across regions beyond Europe and North America.

It should be noted that our study has some limitations. The original search date of this review was August 2023. However, to ensure up-to-date findings, we conducted a search update in September 2024 using the same search strings, the same databases, and the same procedures to identify studies. Additionally, the systematic literature review concentrated solely on studies from North America and Europe, highlighting the need for further research to capture similar studies from regions such as Australia, Asia, and Africa. The limitation of our geographic scope was applied because our findings provide direct input for the health economic evaluation activities performed within the LEGATO project, which currently focuses on Europe, with potential extension to North America. Finally, the study focused on the methodological approach of the identified studies and did not explore the outcomes (ie, cost-benefit ratios) of the included studies.

As a result of our updated search, only a few relevant articles were identified across PubMed, Scopus, Embase, and the Cochrane Library. We identified 1 highly relevant new original study within our geographical focus, tilted “Cost-effectiveness analysis of 11 pharmacotherapies for recurrent glioblastoma in the United States and China.”^44^ This study is important because it investigates patients with recurrent glioblastoma, which was rarely done in the past. This study employed a partitioned survival model to assess the cost-effectiveness of 11 drug treatments, ­including bevacizumab monotherapy, LOM monotherapy, regorafenib monotherapy, nivolumab monotherapy, and 7 distinct bevacizumab-based regimens. The model consisted of 3 health states: progression-free, progression-disease state, and death, with a 5-year time horizon. Clinical data were sourced from a recently published network meta-analysis,^46^ while costs and health outcomes were derived from existing literature. The findings indicated that LOM was the least expensive treatment, whereas regorafenib provided the highest QALYs in both countries. Probabilistic sensitivity analysis showed that LOM was cost-effective, with a probability of over 94%, based on wilingness-to-pay thresholds.^44^

## Conclusion

Although there are numerous publications available on the economic evaluation of glioblastoma, these mostly focus on newly diagnosed glioblastoma patients, thus more studies are needed to investigate the therapies provided to recurrent glioblastoma patients. Despite the huge economic burden on the patients, their family, and caregivers, most studies adopt a health payer perspective. Studies claiming to have a societal perspective were not comprehensive enough, as numerous indirect costs were not accounted for. Our review underscores the urgent need for high-quality and up-to-date quality of life and utility data to support more accurate and comprehensive economic evaluations of glioblastoma treatments.

## Supplementary Material

npaf113_Supplementary_Data

## Data Availability

All the data supporting the findings of this study (ie, the information extracted from the studies included in this review) are available within the article and the Electronic Supplementary Material.
