# Supplementary material for: Methodological aspects of economic evaluations and health economic models in glioblastoma: A systematic literature review
Source: Neurooncol Pract. 2025 Oct 30;13(2):238–51. doi: 10.1093/nop/npaf113 (PMC13153701; doi:10.1093/nop/npaf113)
Supplement: npaf113_Supplementary_Data [file npaf113_supplementary_data.zip › LEGATO_economic_models_supplement_revision2.docx]

# Title page for the supplementary material

**Title**

Methodological aspects of economic evaluations and health economic models in glioblastoma: A systematic literature review

**Authors**

**Erika Országh^1^, Judit Józwiak-Hagymásy^1^, Tamás Dóczi^1^, Dóra Mezei^1^, Bertalan Németh^1^, Hédi Varga^1^, Attila Tordai^2*^, Tomas Kazda^3^, Thierry Gorlia^4^, Caroline Quoilin^4^, Matthias Preusser^5^, Giuseppe Minniti^6^, Marcell Csanádi^1^**

1) Syreon Research Institute, Budapest, Hungary

2) Department of Transfusion Medicine, Semmelweis University, Budapest, Hungary

3) Department of Radiation Oncology, Masaryk Memorial Cancer Institute, Brno, Czech Republic

4) European Organisation for Research and Treatment of Cancer (EORTC), Headquarters, Brussels, Belgium

5) Department of Medicine I, Division of Oncology, Medical University of Vienna, Vienna, Austria

6) Department of Radiological Sciences, Oncology and Anatomical Pathology, Sapienza University of Rome, Rome, Italy

*Corresponding author

Attila Tordai

[tordai.attila@semmelweis.hu](mailto:tordai.attila@semmelweis.hu)

# Appendices

**Appendix I: PICOS criteria to define the research questions**

| **P** (patient/population) | patients with glioma |
| --- | --- |
| **I** (intervention/indicator) | all treatment patterns |
| **C** (comparison) | all treatment patterns |
| **O** (outcomes of interest) | data related to health economic evaluations and models |
| **S** (study design/setting) | - cost-effectiveness analyses - cost-benefit analyses - cost-utility analyses - health economic analyses - health economic evaluations - health technology assessments |

**Appendix II: Systematic literature search strategy and number of hits in Medline, in EMBASE, in SCOPUS, in COCHRANE, and in PROSPERO**

| **MEDLINE** | | | |
| --- | --- | --- | --- |
| **Search No.** | **Concepts** | **Search string** | **Number of hits** |
| #1 | Patient | (glioblastoma*[Title/Abstract]) OR (astrocytoma*[Title/Abstract]) OR (astroglioma*[Title/Abstract]) OR (oligoastrocytoma*[Title/Abstract]) OR (oligodendroglioma*[Title/Abstract]) OR (“low-grade glioma”[Title/Abstract]) OR (“high-grade glioma”[Title/Abstract]) OR (astrocytic[Title/Abstract] AND ( glioma*[Title/Abstract] OR tumor*[Title/Abstract] OR neoplasm*[Title/Abstract])) OR (glioma*[Title/Abstract]) OR (glial[Title/Abstract] AND cell[Title/Abstract] AND tumor*[Title/Abstract]) OR (glial[Title/Abstract] AND (tumor*[Title/Abstract] OR neplasm*[Title/Abstract])) OR (neuroglia*[Title/Abstract] AND (tumor*[Title/Abstract] OR neoplasm*[Title/Abstract])) OR (gliosarcoma*[Title/Abstract]) OR (glyoblastoma*[Title/Abstract]) | 120 395 |
| #2 | Outcome | (cost[Title/Abstract] AND effectiveness[Title/Abstract]) OR cost-effectiveness[Title/Abstract] OR "cost-effectiveness analys*"[Title/Abstract] OR cea[Title/Abstract] OR (cost[Title/Abstract] AND benefit[Title/Abstract]) OR cost-benefit[Title/Abstract] OR "cost-benefit analys*"[Title/Abstract] OR cba[Title/Abstract] OR (cost[Title/Abstract] AND utility[Title/Abstract]) OR cost-utility[Title/Abstract] OR "cost-utility analys*"[Title/Abstract] OR cua[Title/Abstract] OR "incremental cost effectiveness ratio"[Title/Abstract] OR "incremental cost-effectiveness ratio"[Title/Abstract] OR icer[Title/Abstract] OR "discrete event simulation"[Title/Abstract] OR markov[Title/Abstract] OR "decision tree"[Title/Abstract] OR (economic[Title/Abstract] AND (model*[Title/Abstract] OR analys*[Title/Abstract] OR evaluation[Title/Abstract])) OR "health technology assessment"[Title/Abstract] OR hta[Title/Abstract] OR ((health[Title/Abstract] AND economic[Title/Abstract]) AND (model*[Title/Abstract] OR analys*[Title/Abstract] OR evaluation[Title/Abstract])) OR "event history model"[Title/Abstract] OR microsimulation[Title/Abstract] OR "Monte Carlo"[Title/Abstract] OR "Monte-Carlo"[Title/Abstract] OR "patient level simulation"[Title/Abstract] OR "patient-level simulation"[Title/Abstract] OR "deterministic sensitivity analys*"[Title/Abstract] OR "probabilistic sensitivity analys*"[Title/Abstract] OR "simulation model*"[Title/Abstract] OR "transition probabilit*"[Title/Abstract] OR "Tornado diagram"[Title/Abstract] | 417 957 |
| **#3** | **Combined search** | **#1 AND #2** | **625** |
| **#4** | **Filters** | **Limit to English** | **604** |

| **EMBASE** | | | |
| --- | --- | --- | --- |
| **Search No.** | **Concepts** | **Search string** | **Number of hits** |
| #1 | Patient | glioblastoma*:ti,ab,kw OR astrocytoma*:ti,ab,kw OR astroglioma*:ti,ab,kw OR oligoastrocytoma*:ti,ab,kw OR oligodendroglioma*:ti,ab,kw OR (low-grade glioma:ti,ab,kw) OR (high-grade glioma:ti,ab,kw) OR (astrocytic:ti,ab,kw AND (glioma*:ti,ab,kw OR tumor*:ti,ab,kw OR neoplasm*:ti,ab,kw)) OR glioma*:ti,ab,kw OR (glial:ti,ab,kw AND cell:ti,ab,kw AND tumor*:ti,ab,kw) OR (glial:ti,ab,kw AND (tumor*:ti,ab,kw OR neplasm*:ti,ab,kw)) OR (neuroglia*:ti,ab,kw AND (tumor*:ti,ab,kw OR neoplasm*:ti,ab,kw)) OR gliosarcoma*:ti,ab,kw OR glyoblastoma*:ti,ab,kw | 170 657 |
| #2 | Outcome | cost:ti,ab,kw AND effectiveness:ti,ab,kw OR 'cost effectiveness':ti,ab,kw OR 'cost-effectiveness analys*':ti,ab,kw OR cea:ti,ab,kw OR (cost:ti,ab,kw AND benefit:ti,ab,kw) OR 'cost benefit':ti,ab,kw OR 'cost-benefit analys*':ti,ab,kw OR cba:ti,ab,kw OR (cost:ti,ab,kw AND utility:ti,ab,kw) OR 'cost utility':ti,ab,kw OR 'cost-utility analys*':ti,ab,kw OR cua:ti,ab,kw OR 'incremental cost effectiveness ratio':ti,ab,kw OR 'incremental cost-effectiveness ratio':ti,ab,kw OR icer:ti,ab,kw OR 'discrete event simulation':ti,ab,kw OR markov:ti,ab,kw OR 'decision tree':ti,ab,kw OR (economic:ti,ab,kw AND (model*:ti,ab,kw OR analys*:ti,ab,kw OR evaluation:ti,ab,kw)) OR 'health technology assessment':ti,ab,kw OR hta:ti,ab,kw OR (health:ti,ab,kw AND economic:ti,ab,kw AND (model*:ti,ab,kw OR analys*:ti,ab,kw OR evaluation:ti,ab,kw)) OR 'event history model':ti,ab,kw OR microsimulation:ti,ab,kw OR 'monte carlo':ti,ab,kw OR 'monte-carlo':ti,ab,kw OR 'patient level simulation':ti,ab,kw OR 'patient-level simulation':ti,ab,kw OR | 536 984 |
| **#3** | **Combined search** | **#1 AND #2** | **1 038** |
| **#4** | **Filters** | **Limit to English** | **1 008** |

| **SCOPUS** | | | |
| --- | --- | --- | --- |
| **Search No.** | **Concepts** | **Search string** | **Number of hits** |
| #1 | Patient | TITLE-ABS-KEY ( ( glioblastoma* ) OR ( astrocytoma* ) OR ( astroglioma* ) OR ( oligoastrocytoma* ) OR ( oligodendroglioma* ) OR ( low-grade glioma ) OR ( high-grade glioma ) OR ( astrocytic AND ( glioma* OR tumor* OR neoplasm* ) ) OR ( glioma* ) OR ( glial AND cell AND tumor* ) OR ( glial AND ( tumor* OR neplasm* ) ) OR ( neuroglia* AND ( tumor* OR neoplasm* ) ) OR ( gliosarcoma* ) OR ( glyoblastoma* ) ) | 185 412 |
| #2 | Outcome | TITLE-ABS-KEY((cost and effectiveness) or cost-effectiveness or "cost-effectiveness analys*" or cea or (cost and benefit) or cost-benefit or "cost-benefit analys*" or cba or (cost and utility) or cost-utility or "cost-utility analys*" or cua or "incremental cost effectiveness ratio" or "incremental cost-effectiveness ratio" or icer or "discrete event simulation" or markov or "decision tree" or (economic and (model* or analys* or evaluation)) or "health technology assessment" or hta or ((health and economic) and (model* or analys* or evaluation)) or "event history model" or microsimulation or "Monte Carlo" or "Monte-Carlo" or "patient level simulation" or "patient-level simulation" or "deterministic sensitivity analys*" or "probabilistic sensitivity analys*" or "simulation model*" or "transition probabilit*" or "Tornado diagram") | 2 845 060 |
| **#3** | **Combined search** | **#1 AND #2** | **2 051** |
| **#4** | **Filters** | **Limit to English** | **1 993** |

| **COCHRANE** | | | |
| --- | --- | --- | --- |
| **Search No.** | **Concepts** | **Search string** | **Number of hits** |
| #1 | Patient | (glioblastoma*) OR (astrocytoma*) OR (astroglioma*) OR (oligoastrocytoma*) OR (oligodendroglioma*) OR (low-grade glioma) OR (high-grade glioma) OR (astrocytic AND ( glioma* OR tumor* OR neoplasm*)) OR (glioma*) OR (glial AND cell AND tumor*) OR (glial AND (tumor* OR neplasm*)) OR (neuroglia* AND (tumor* OR neoplasm*)) OR (gliosarcoma*) OR (glyoblastoma*) in Title Abstract Keyword | 26 reviews,  4 protocols |

| **PROSPERO** | | | |
| --- | --- | --- | --- |
| **Search No.** | **Concepts** | **Search string** | **Number of hits** |
| #1 | Patient | (glioblastoma*) OR (astrocytoma*) OR (astroglioma*) OR (oligoastrocytoma*) OR (oligodendroglioma*) OR (low-grade glioma) OR (high-grade glioma) OR (astrocytic AND ( glioma* OR tumor* OR neoplasm*)) OR (glioma*) OR (glial AND cell AND tumor*) OR (glial AND (tumor* OR neplasm*)) OR (neuroglia* AND (tumor* OR neoplasm*)) OR (gliosarcoma*) OR (glyoblastoma*) | 564 |
| #2 | Outcome | (cost and effectiveness) or cost-effectiveness or "cost-effectiveness analys*" or cea or (cost and benefit) or cost-benefit or "cost-benefit analys*" or cba or (cost and utility) or cost-utility or "cost-utility analys*" or cua or "incremental cost effectiveness ratio" or "incremental cost-effectiveness ratio" or icer or "discrete event simulation" or markov or "decision tree" or (economic and (model* or analys* or evaluation)) or "health technology assessment" or hta or ((health and economic) and (model* or analys* or evaluation)) or "event history model" or microsimulation or "Monte Carlo" or "Monte-Carlo" or "patient level simulation" or "patient-level simulation" or "deterministic sensitivity analys*" or "probabilistic sensitivity analys*" or "simulation model*" or "transition probabilit*" or "Tornado diagram" | 22 395 |
| **#3** | **Combined search** | **#1 AND #2** | **38** |

**Appendix III: Eligibility criteria used to select relevant publications**

|  | **Criteria** | **Inclusion** | **Exclusion** |
| --- | --- | --- | --- |
| **PICO(S)** | Population/participants | Adult patients with diffuse glioma | No eligibility restrictions |
|  | Interventions | All treatment patterns | No eligibility restrictions |
|  | Comparators | All treatment patterns | No eligibility restrictions |
|  | Outcomes | Data related to health economics models | No eligibility restrictions |
|  | Study design/Setting | - cost-effectiveness analyses - cost-benefit analyses - cost-utility analyses - health economic analyses - health economic evaluations - health technology assessments | Not original studies were excluded |
| **Other eligibility criteria** | Geographical location | Europe and North America | Studies with other focus than Europe and North-America were excluded |
|  | Study period | No publication date limit | No exclusion criteria |
|  | Language criteria | Full text studies written in English were eligible for data extraction. | Full text studies written in any other language than English were not eligible for data collection.  All articles with irrelevant title and without English abstract were excluded. |

**Appendix IV: Data items to be collected**

| Data domain | Data collected |
| --- | --- |
| Paper identification | - unique ID |
| General information | - first author - publication year - paper title - journal - DOI number - research question / aim / objective |
| Health economic model information | - number of models in the article - type of economic evaluation (e.g., CEA, CUA) - additional comments related to the type of economic evaluation - country (the model was adapted to) - patient population - comorbidity (description of simulating comorbidities) - adverse events (description of simulating adverse events) - perspective - if societal perspective was used, which type of additional measurements were added? - model type/design - additional comments related to the model type - source of original model (if adapted) - model arms - list of health states (if applicable) - model structure - model structure figure - time horizon - cycle length (if applicable) - discount rate(s) - currency - reference year for costs - transition data - transition probability data table in the article - data source of transition data - health gain data (e.g., utility) - health gain data table in the article - data source of health gain data - cost data - cost data table in the article - data source of cost data - validation method (Additional information about the validation method) - sensitivity analysis and/or scenario analysis (What kind of scenario analysis / sensitivity analysis was performed?) |

CEA: Cost-effectiveness analysis, CUA: cost-utility analysis

**Appendix V: List of grey literature sources**

To find relevant health economic evaluations and models, the following webpages of health technology assessment/appraisal agencies and conference abstracts were searched:

- Agency for Healthcare Research and Quality (AHRQ)
- Canadian Agency for Drug and Technologies in Health (CADTH)
- National Institute for Health and Care Excellence (NICE)
- Medical Services Advisory Committee (MSAC)
- Ludwig Boltzmann Institut für Health Technology Assessment (LBI)
- Belgian Health Care Knowledge Centre (KCE)
- Danish Health and Medicines Authority (DHMA)
- French National Authority for Health (HAS)
- Institut für Qualität und Wirtschaftlichkeit im Gesundheitswesen (IQWiG)
- National Centre for Pharmacoeconomics, Ireland (NCPE)
- National Health Care Institute Netherlands
- Norwegian Institute of Public Health (NIPH)
- Swedish Agency for Health Technology Assessment (SBU)
- ISPOR abstracts via Value in Health
- other conference abstracts via Embase

**Appendix VI: Economic evaluations without using modelling methods**

There were 8 studies ^1-8^, which used simple calculation of costs and benefits without applying economic models. These are only briefly described here. Two studies investigated the cost and benefits of radiotherapy in different treatment regiments ^1-2^. Two studies calculated the costs and outcomes of temozolomide ^3-4.^ One study investigated modulated electrohyperthermia ^5^, another one calculated the cost and benefits of the bevacizumab+irinotecan (BVZ/CPT-11) combination ^6^. One study evaluated the treatment of unmethylated MGMT-promoter recurrent glioblastoma with cancer stem cell assay-guided chemotherapy and the impact on patients’ healthcare costs ^7^. There was one study where the authors compared the pattern of usual care of patients with glioblastoma in a French hospital and aimed to determine the cost-effectiveness of novel therapeutic strategies (combining radiotherapy plus temozolomide and the use of bevacizumab at recurrence) ^8^. All studies performed a cost-effectiveness analysis, where the incremental cost per life-year gain/saved was calculated. In two studies cost-utility analysis was also performed and the incremental cost / QALY gained was also expressed ^2, 5^. In five studies the patients had newly diagnosed glioblastoma ^1-4, 8^, while in three studies they had recurrent glioblastoma ^5-7^.

Corresponding references:

1 Norum J. Radiotherapy costs in glioblastoma. Oncol Rep. 1996.

2 Ghosh S, Baker S, De Castro DG, et al. Improved cost-effectiveness of short-course radiotherapy in elderly and/or frail patients with glioblastoma. Radiother Oncol. 2018;127(1):114–20.

3 Krysanov I, Krysanova V. Pharmacoeconomic analysis of intravenous temozolomide for the treatment of newly diagnosed glioblastoma multiforme in Russia. Value Health. 2013; 16(7):A413.

4 Lamers LM, Stupp R, Van Den Bent MJ, et al. Cost‐effectiveness of temozolomide for the treatment of newly diagnosed glioblastoma multiforme: A report from the EORTC 26981/22981 NCI‐C CE3 Intergroup Study. Cancer, 2008;112(6):1337–44.

5 Roussakow SV. Clinical and economic evaluation of modulated electrohyperthermia concurrent to dose-dense temozolomide 21/28 days regimen in the treatment of recurrent glioblastoma: A retrospective analysis of a two-centre German cohort trial with systematic comparison and effect-to-treatment analysis. BMJ Open. 2017;7(11):e017387.

6 Ruiz-Sánchez D, Peinado II, Alaguero-Calero M, Sastre-Heres AJ, Diez BG, Peña-Díaz J. Cost-effectiveness analysis of the bevacizumab-irinotecan regimen in the treatment of primary glioblastoma multiforme recurrences. Oncology Letters. 2016;12(3):1935–40.

7 Ranjan T, Yu A, Elhamdani S, et al. Treatment of unmethylated MGMT-promoter recurrent glioblastoma with cancer stem cell assay-guided chemotherapy and the impact on patients’ healthcare costs. Neuro-Oncol Adv. 2023;5(1):vdad055.

8 Diebold G, Ducray F, Henaine AM, et al. Management of glioblastoma: Comparison of clinical practices and cost-effectiveness in two cohorts of patients (2008 versus 2004) diagnosed in a French university hospital. J Clin Pharm Ther. 2014;39(6):642–8.

**Appendix VII: Results of quality assessment on economic evaluation studies on pharmaceutical therapies in combination with radiotherapy**

| Type of bias | Issues addressed | **Chen, 2021** | | **Connock, 2021** | | **Fisher, 2016** | | **Kovic, 2015** | | **Messali, 2013** | | **Waschke, 2018** | |
| --- | --- | --- | --- | --- | --- | --- | --- | --- | --- | --- | --- | --- | --- |
|  |  | Relevant to study Yes/ No/ Partly/ Unclear/ NA | How did you deal with this bias?  (description of strategy and rationale) | Relevant to study Yes/ No/ Partly/ Unclear/ NA | How did you deal with this bias?  (description of strategy and rationale) | Relevant to study Yes/ No/ Partly/ Unclear/ NA | How did you deal with this bias?  (description of strategy and rationale) | Relevant to study Yes/ No/ Partly/ Unclear/ NA | How did you deal with this bias?  (description of strategy and rationale) | Relevant to study Yes/ No/ Partly/ Unclear/ NA | How did you deal with this bias?  (description of strategy and rationale) | Relevant to study Yes/ No/ Partly/ Unclear/ NA | How did you deal with this bias?  (description of strategy and rationale) |
| PART A. Overall checklist for bias in economic evaluation | | | | | | | | | | | | | |
| Narrow perspective bias | Was a societal perspective adopted? If not, has a different perspective been justified? | Yes | Health care system | Yes | 1) Societal 2) Health care system | Yes | Health care system | Yes | Health care system | Yes | Societal | Yes | Health care system |
| Inefficient comparator bias | Was the best alternative chosen as comparator? Was current practice chosen as a comparator? Have all comparators been described in sufficient detail? | Yes |  | Yes |  | Yes |  | Yes |  | Yes |  | Yes |  |
| Cost measurement omission bias | Were all costs relevant to the disease and intervention identified and considered? | Yes |  | Yes |  | Yes |  | Yes |  | Yes |  | Yes |  |
| Intermittent data collection bias | Was the resource use measured continuously? | Yes |  | Yes |  | Yes |  | Yes |  | Yes |  | Yes |  |
| Invalid valuation bias | Is the price calculation presented in a detailed manner? Have reference prices been used? | Yes |  | Yes |  | Yes |  | Yes |  | Yes |  | Yes |  |
| Ordinal ICER bias | Have cardinal scales for the outcomes measure in a CEA been used? | Yes | ∆ Cost / ∆ QALY | Yes | ∆ Cost / ∆ QALY | Yes | ∆ Cost / ∆ QALY | Yes | ∆ Cost / ∆ QALY | Yes | ∆ Cost / ∆ QALY | Yes | ∆ Cost / ∆ quality-adjusted life-month  ∆ Cost / ∆ QALY |
| Double-counting bias | Are variables adequately checked for double-counting? | Yes |  | Yes |  | Yes |  | Yes |  | Yes |  | Yes |  |
| Inappropriate discounting bias | Have discounting rates from guidelines been applied? | Yes | 3.0% | Yes | 1) 3.0% 2) 5.0% | Yes | 5.0% | Yes | 5.0% | Yes | 3.0% | No | No discounting mentioned |
| Limited sensitivity analysis bias | Have the four principles of uncertainty (methodological, structural, heterogeneity, parameter) been considered in sufficient detail? | Yes | DSA and PSA | Yes | DSA and PSA | Yes | DSA and PSA | Yes | DSA and PSA | Yes | DSA and PSA | Yes | DSA |
| Sponsor bias | Have sponsorships been disclosed? Is the study protocol freely accessible? | Yes |  | No | No statement provided by the authors. | Yes |  | Yes |  | Yes |  | Yes |  |
| Reporting and dissemination bias | Has the study/trial been listed in a trial register? Have all results been reported according to the study protocol? | NA |  | NA |  | NA |  | NA |  | NA |  | NA |  |
| PART B. Model-specific aspects of bias in economic evaluation | | | | | | | | | | | | | |
| I Bias related to structure | | | | | | | | | | | | | |
| Structural assumptions bias | Is the model structure in line with coherent theory? Do treatment pathways reflect the nature of disease? | Yes |  | Yes |  | Yes |  | Yes |  | Yes |  | Yes |  |
| No treatment comparator bias | Is there an adequate comparator, i.e. care as usual? | Yes |  | Yes |  | Yes |  | Yes |  | Yes |  | Yes |  |
| Wrong model bias | Is the model chosen adequate regarding the decision problem? | Yes |  | Yes |  | Yes |  | Yes |  | Yes |  | Yes |  |
| Limited time horizon bias | Was a lifetime horizon chosen? Were shorter time horizons adequately justified? | Yes | 5 years | Yes | 1) 5 years 2) 2 years | Yes | 5 years | Yes | 2 years | Yes | 5 years | Yes | 5 years |
| II Bias related to data | | | | | | | | | | | | | |
| Bias related to data identification | Are the methods of data identification transparent? Are all choices justified adequately? Do the input parameters come from highquality and well-designed studies? | Yes |  | Yes |  | Yes |  | Yes |  | Yes |  | Yes |  |
| Bias related to baseline data | Are probabilities, for example, based on natural history data? Is transformation of rates into transition probabilities done accurately? | Yes |  | Yes |  | Yes |  | Yes |  | Yes |  | Yes |  |
| Bias related to treatment effects | Are relative treatment effects synthesized using appropriate metaanalytic techniques? Are extrapolations documented and well justified? Are alternative assumptions explored regarding extrapolation? | Yes |  | Yes |  | Yes |  | Yes |  | Yes |  | Yes |  |
| Bias related to quality of life weights (utilities) | Are the utilities incorporated appropriate for the specific decision problem? | Yes |  | Yes |  | Yes |  | Yes |  | Yes |  | Yes |  |
| Non-transparent data incorporation bias | Is the process of data incorporation transparent? Are all data and their sources described in detail? | Yes |  | Yes |  | Yes |  | Yes |  | Yes |  | Yes |  |
| Limited scope bias | Have the four principles of uncertainty (methodological, structural, heterogeneity, parameter) been considered? | Yes |  | Yes |  | Yes |  | Yes |  | Yes |  | Yes |  |
| III Bias related to consistency | | | | | | | | | | | | | |
| Bias related to internal consistency | Has internal consistency in terms of mathematical logic been evaluated? | Unclear |  | Unclear |  | Unclear |  | Unclear |  | Unclear |  | Unclear |  |

**Appendix VIII: Results of quality assessment on economic evaluations on tumour treating fields in combination with different therapies**

| Type of bias | Issues addressed | **Bernard-Arnoux, 2016** | | **Connock, 2019** | | **Connock, 2021** | | **Nino de Rivera, 2023** | | **Guzauskas, 2019** | |
| --- | --- | --- | --- | --- | --- | --- | --- | --- | --- | --- | --- |
|  |  | Relevant to study Yes/ No/ Partly/ Unclear/ NA | How did you deal with this bias?  (description of strategy and rationale) | Relevant to study Yes/ No/ Partly/ Unclear/ NA | How did you deal with this bias?  (description of strategy and rationale) | Relevant to study Yes/ No/ Partly/ Unclear/ NA | How did you deal with this bias?  (description of strategy and rationale) | Relevant to study Yes/ No/ Partly/ Unclear/ NA | How did you deal with this bias?  (description of strategy and rationale) | Relevant to study Yes/ No/ Partly/ Unclear/ NA | How did you deal with this bias?  (description of strategy and rationale) |
| Narrow perspective bias | Was a societal perspective adopted? If not, has a different perspective been justified? | Yes | Health care system | Yes | Health care system | Yes | Health care system | Yes | Health care system | Yes | Health care system |
| Inefficient comparator bias | Was the best alternative chosen as comparator? Was current practice chosen as a comparator? Have all comparators been described in sufficient detail? | Yes |  | Yes |  | Yes |  | Yes |  | Yes |  |
| Cost measurement omission bias | Were all costs relevant to the disease and intervention identified and considered? | Yes |  | Yes |  | Yes |  | Yes |  | Yes |  |
| Intermittent data collection bias | Was the resource use measured continuously? | Yes |  | Yes |  | Yes |  | Yes |  | Yes |  |
| Invalid valuation bias | Is the price calculation presented in a detailed manner? Have reference prices been used? | Yes |  | Yes |  | Yes |  | Yes |  | Yes |  |
| Ordinal ICER bias | Have cardinal scales for the outcomes measure in a CEA been used? | Yes | ∆ Cost / ∆ LYG | Yes | ∆ Cost / ∆ LYG; ∆ Cost / ∆ QALY | No | ∆ Cost / ∆ LYG | Yes | ∆ Cost / ∆ LY, ∆ Cost / ∆ QALY | Yes | ∆ Cost / ∆ LYG, ∆ Cost / ∆ QALY |
| Double-counting bias | Are variables adequately checked for double-counting? | Yes |  | Yes |  | Yes |  | No | The analysis does not provide evidence that variables were checked for double-counting. | Yes |  |
| Inappropriate discounting bias | Have discounting rates from guidelines been applied? | Yes | 4.0% | Yes | 4.0% | Yes | 4.0% | Yes | 2.5% | Yes | 3.0% |
| Limited sensitivity analysis bias | Have the four principles of uncertainty (methodological, structural, heterogeneity, parameter) been considered in sufficient detail? | Yes | DSA and PSA | Yes | DSA and PSA | Yes | DSA and PSA | Yes | DSA and PSA | Yes | DSA and PSA |
| Sponsor bias | Have sponsorships been disclosed? Is the study protocol freely accessible? | Yes |  | Yes |  | No | No statement provided by the authors. | Yes |  | Yes |  |
| Reporting and dissemination bias | Has the study/trial been listed in a trial register? Have all results been reported according to the study protocol? | NA |  | NA |  | NA |  | NA |  | NA |  |
| Structural assumptions bias | Is the model structure in line with coherent theory? Do treatment pathways reflect the nature of disease? | Yes |  | Yes |  | Yes |  | Yes |  | Yes |  |
| No treatment comparator bias | Is there an adequate comparator, i.e. care as usual? | Yes |  | Yes |  | Yes |  | Yes |  | Yes |  |
| Wrong model bias | Is the model chosen adequate regarding the decision problem? | Yes |  | Yes |  | Yes |  | Yes |  | Yes |  |
| Limited time horizon bias | Was a lifetime horizon chosen? Were shorter time horizons adequately justified? | Yes | Lifetime | NA | 20 years | Yes | 12.5 years | NA | 20 years | Yes | Lifetime |
| Bias related to data identification | Are the methods of data identification transparent? Are all choices justified adequately? Do the input parameters come from highquality and well-designed studies? | Yes |  | Yes |  | Yes |  | Yes |  | Yes |  |
| Bias related to baseline data | Are probabilities, for example, based on natural history data? Is transformation of rates into transition probabilities done accurately? | Yes |  | Yes |  | Yes |  | Yes |  | Yes |  |
| Bias related to treatment effects | Are relative treatment effects synthesized using appropriate metaanalytic techniques? Are extrapolations documented and well justified? Are alternative assumptions explored regarding extrapolation? | Yes |  | Yes |  | Yes |  | Yes |  | Yes |  |
| Bias related to quality of life weights (utilities) | Are the utilities incorporated appropriate for the specific decision problem? | NA | No utilities incorporated in the model. | Yes |  | NA | No utilities incorporated in the model. | Yes |  | Yes |  |
| Non-transparent data incorporation bias | Is the process of data incorporation transparent? Are all data and their sources described in detail? | Yes |  | Yes |  | Yes |  | Yes |  | Yes |  |
| Limited scope bias | Have the four principles of uncertainty (methodological, structural, heterogeneity, parameter) been considered? | Yes |  | Yes |  | Yes |  | Yes |  | Yes |  |
| Bias related to internal consistency | Has internal consistency in terms of mathematical logic been evaluated? | Unclear |  | Unclear |  | Unclear |  | Unclear |  | Unclear |  |

**Appendix IX: Results of quality assessment on economic evaluation studies on pharmaceutical therapies in recurrent glioblastoma**

| Type of bias | Issues addressed | **Garcia Lopez, 2014** | | **Martikainen, 2005** | |
| --- | --- | --- | --- | --- | --- |
|  |  | Relevant to study Yes/ No/ Partly/ Unclear/ NA | How did you deal with this bias?  (description of strategy and rationale) | Relevant to study Yes/ No/ Partly/ Unclear/ NA | How did you deal with this bias?  (description of strategy and rationale) |
| Narrow perspective bias | Was a societal perspective adopted? If not, has a different perspective been justified? | Yes | Health care system | Partly | A societal perspective was assumed, cost of travelling per visit was taken into account. |
| Inefficient comparator bias | Was the best alternative chosen as comparator? Was current practice chosen as a comparator? Have all comparators been described in sufficient detail? | Yes |  | Yes |  |
| Cost measurement omission bias | Were all costs relevant to the disease and intervention identified and considered? | Yes |  | Yes |  |
| Intermittent data collection bias | Was the resource use measured continuously? | Yes |  | Yes |  |
| Invalid valuation bias | Is the price calculation presented in a detailed manner? Have reference prices been used? | Yes |  | Yes |  |
| Ordinal ICER bias | Have cardinal scales for the outcomes measure in a CEA been used? | Yes | ∆ Cost / ∆ year to obtain 6 months PFS with stable health state utility value | Yes | ∆ Cost / ∆ life-month, ∆ Cost / ∆ progression-free months, ∆ Cost / ∆ QALY |
| Double-counting bias | Are variables adequately checked for double-counting? | Yes |  | Yes |  |
| Inappropriate discounting bias | Have discounting rates from guidelines been applied? | Yes | 3.0% | Yes | 5.0% |
| Limited sensitivity analysis bias | Have the four principles of uncertainty (methodological, structural, heterogeneity, parameter) been considered in sufficient detail? | Yes | DSA and PSA | Yes | PSA and other |
| Sponsor bias | Have sponsorships been disclosed? Is the study protocol freely accessible? | Yes |  | Yes |  |
| Reporting and dissemination bias | Has the study/trial been listed in a trial register? Have all results been reported according to the study protocol? | NA |  | NA |  |
| Structural assumptions bias | Is the model structure in line with coherent theory? Do treatment pathways reflect the nature of disease? | Yes |  | Yes |  |
| No treatment comparator bias | Is there an adequate comparator, i.e. care as usual? | Yes |  | Yes |  |
| Wrong model bias | Is the model chosen adequate regarding the decision problem? | Yes |  | Yes |  |
| Limited time horizon bias | Was a lifetime horizon chosen? Were shorter time horizons adequately justified? | Yes | The time horizon of 1 year is appropriate, considering the disease's prognosis. | Yes | Lifetime |
| Bias related to data identification | Are the methods of data identification transparent? Are all choices justified adequately? Do the input parameters come from highquality and well-designed studies? | Yes |  | Yes |  |
| Bias related to baseline data | Are probabilities, for example, based on natural history data? Is transformation of rates into transition probabilities done accurately? | Yes |  | Yes |  |
| Bias related to treatment effects | Are relative treatment effects synthesized using appropriate metaanalytic techniques? Are extrapolations documented and well justified? Are alternative assumptions explored regarding extrapolation? | Yes |  | Yes |  |
| Bias related to quality of life weights (utilities) | Are the utilities incorporated appropriate for the specific decision problem? | Yes |  | Partly | QOL estimates were gathered using proxy respondents. Utility scores were obtained using a visual analogue scale (VAS) method. The questionnaires were sent to eight leading neuro-oncologists, of whom six responded. |
| Non-transparent data incorporation bias | Is the process of data incorporation transparent? Are all data and their sources described in detail? | Unclear | Some data source is unclear. | Yes |  |
| Limited scope bias | Have the four principles of uncertainty (methodological, structural, heterogeneity, parameter) been considered? | Yes |  | Yes |  |
| Bias related to internal consistency | Has internal consistency in terms of mathematical logic been evaluated? | Unclear |  | Unclear |  |

**Appendix X: Results of quality assessment on economic evaluation studies on other types of interventions**

| Type of bias | Issues addressed | **Baguet, 2019** | | **Chandra, 2019** | |
| --- | --- | --- | --- | --- | --- |
|  |  | Relevant to study Yes/ No/ Partly/ Unclear/ NA | How did you deal with this bias?  (description of strategy and rationale) | Relevant to study Yes/ No/ Partly/ Unclear/ NA | How did you deal with this bias?  (description of strategy and rationale) |
| Narrow perspective bias | Was a societal perspective adopted? If not, has a different perspective been justified? | Yes | Health care system | Yes | Hospital. |
| Inefficient comparator bias | Was the best alternative chosen as comparator? Was current practice chosen as a comparator? Have all comparators been described in sufficient detail? | Yes |  | Partly | Using insurance type as a comparator does not align with standard practices for economic evaluations, but is acceptable nonetheless. |
| Cost measurement omission bias | Were all costs relevant to the disease and intervention identified and considered? | Yes |  | Yes |  |
| Intermittent data collection bias | Was the resource use measured continuously? | Yes |  | Yes |  |
| Invalid valuation bias | Is the price calculation presented in a detailed manner? Have reference prices been used? | Yes |  | Yes |  |
| Ordinal ICER bias | Have cardinal scales for the outcomes measure in a CEA been used? | Yes | ∆ Cost / ∆ identified non-responder to follow-up treatment | Yes | ∆ Cost / ∆ QALY |
| Double-counting bias | Are variables adequately checked for double-counting? | Yes |  | Yes |  |
| Inappropriate discounting bias | Have discounting rates from guidelines been applied? | NA | As the analysis focused on a short time horizon, discounting was not considered relevant. | NA | Discounting was not relevant for the analysis focused on a short-term hospital stay. |
| Limited sensitivity analysis bias | Have the four principles of uncertainty (methodological, structural, heterogeneity, parameter) been considered in sufficient detail? | Yes | DSA and PSA | No | The study did not conduct any sensitivity analyses to explore the impact of uncertainty. |
| Sponsor bias | Have sponsorships been disclosed? Is the study protocol freely accessible? | Yes |  | Yes |  |
| Reporting and dissemination bias | Has the study/trial been listed in a trial register? Have all results been reported according to the study protocol? | NA |  | NA |  |
| Structural assumptions bias | Is the model structure in line with coherent theory? Do treatment pathways reflect the nature of disease? | Yes |  | NA | Since no formal economic model was used, assessing structural assumptions does not apply. |
| No treatment comparator bias | Is there an adequate comparator, i.e. care as usual? | Yes |  | Yes | Using insurance type as a comparator does not align with standard practices for economic evaluations, but is acceptable nonetheless. |
| Wrong model bias | Is the model chosen adequate regarding the decision problem? | Yes |  | NA | The study's design did not involve the use of a formal model. |
| Limited time horizon bias | Was a lifetime horizon chosen? Were shorter time horizons adequately justified? | NA | Not applicable for this type of evaluation. | NA | Not applicable for this type of evaluation. |
| Bias related to data identification | Are the methods of data identification transparent? Are all choices justified adequately? Do the input parameters come from highquality and well-designed studies? | Yes |  | Yes |  |
| Bias related to baseline data | Are probabilities, for example, based on natural history data? Is transformation of rates into transition probabilities done accurately? | Yes |  | Yes |  |
| Bias related to treatment effects | Are relative treatment effects synthesized using appropriate metaanalytic techniques? Are extrapolations documented and well justified? Are alternative assumptions explored regarding extrapolation? | Yes |  | NA | The assessment of treatment effects is not applicable since the study did not compare clinical outcomes based on specific treatments. |
| Bias related to quality of life weights (utilities) | Are the utilities incorporated appropriate for the specific decision problem? | NA | The study focused on diagnostic cost-effectiveness and did not incorporate utility weights. | Yes |  |
| Non-transparent data incorporation bias | Is the process of data incorporation transparent? Are all data and their sources described in detail? | Yes |  | Yes |  |
| Limited scope bias | Have the four principles of uncertainty (methodological, structural, heterogeneity, parameter) been considered? | Yes |  | No | The study did not conduct any sensitivity analyses to explore the impact of uncertainty. |
| Bias related to internal consistency | Has internal consistency in terms of mathematical logic been evaluated? | Unclear |  | Unclear |  |
